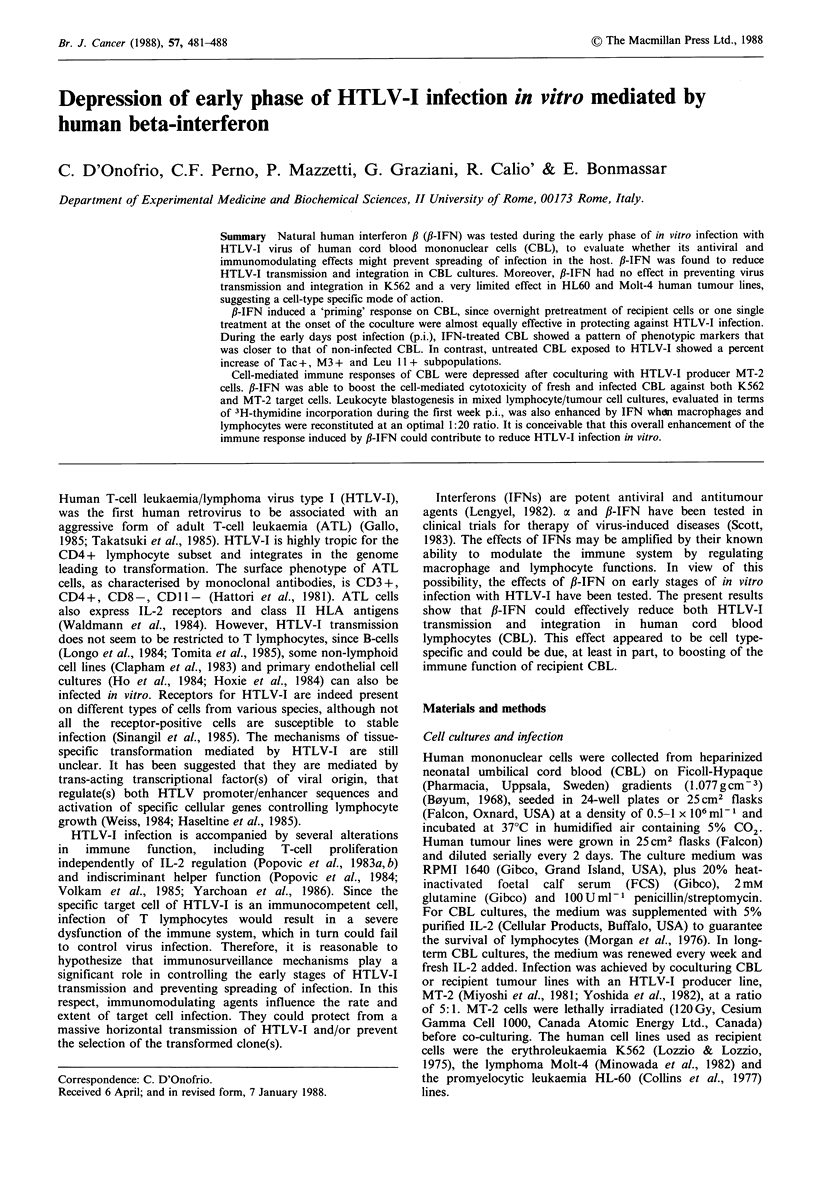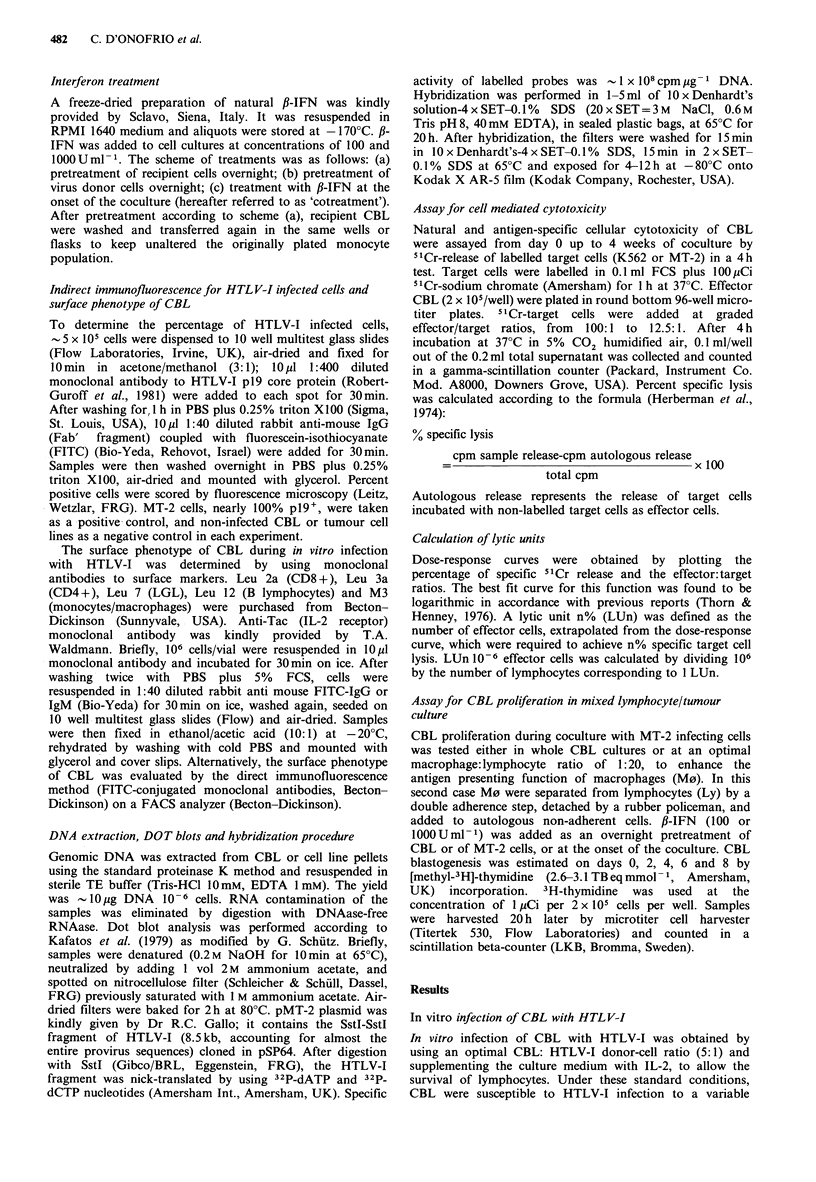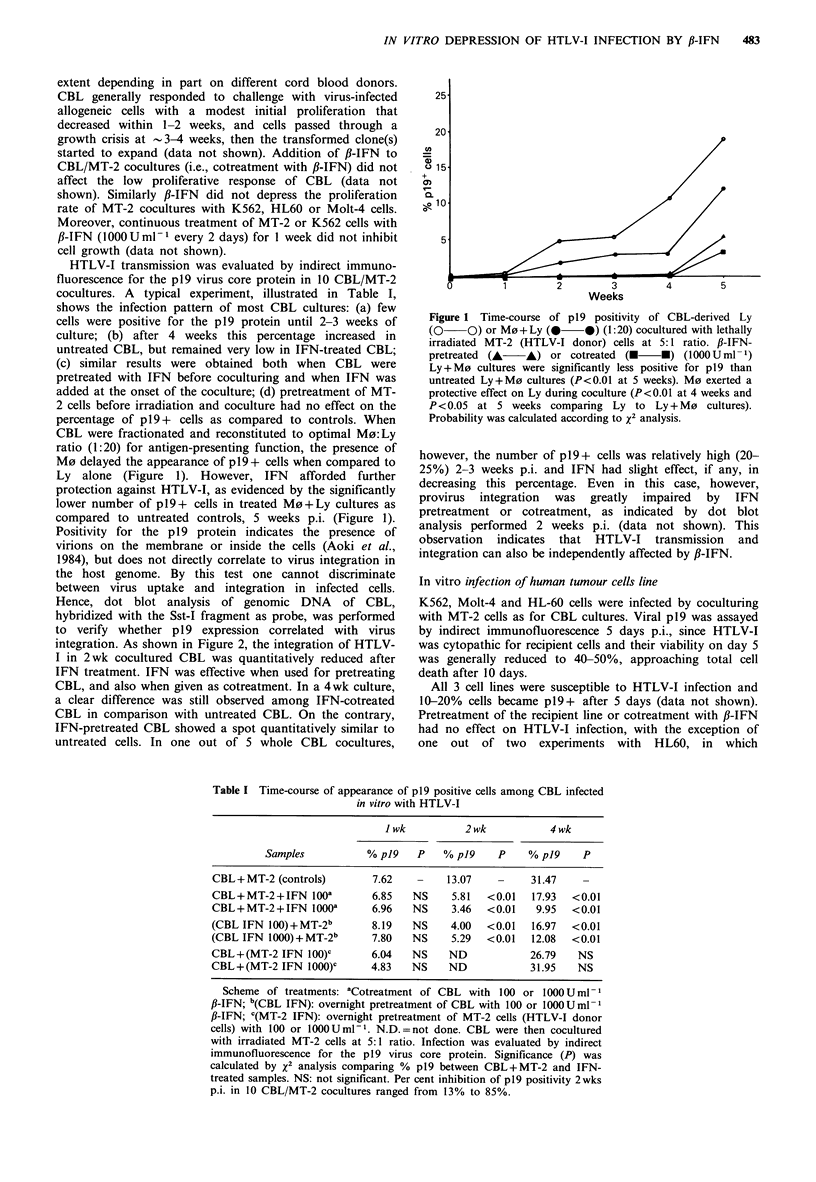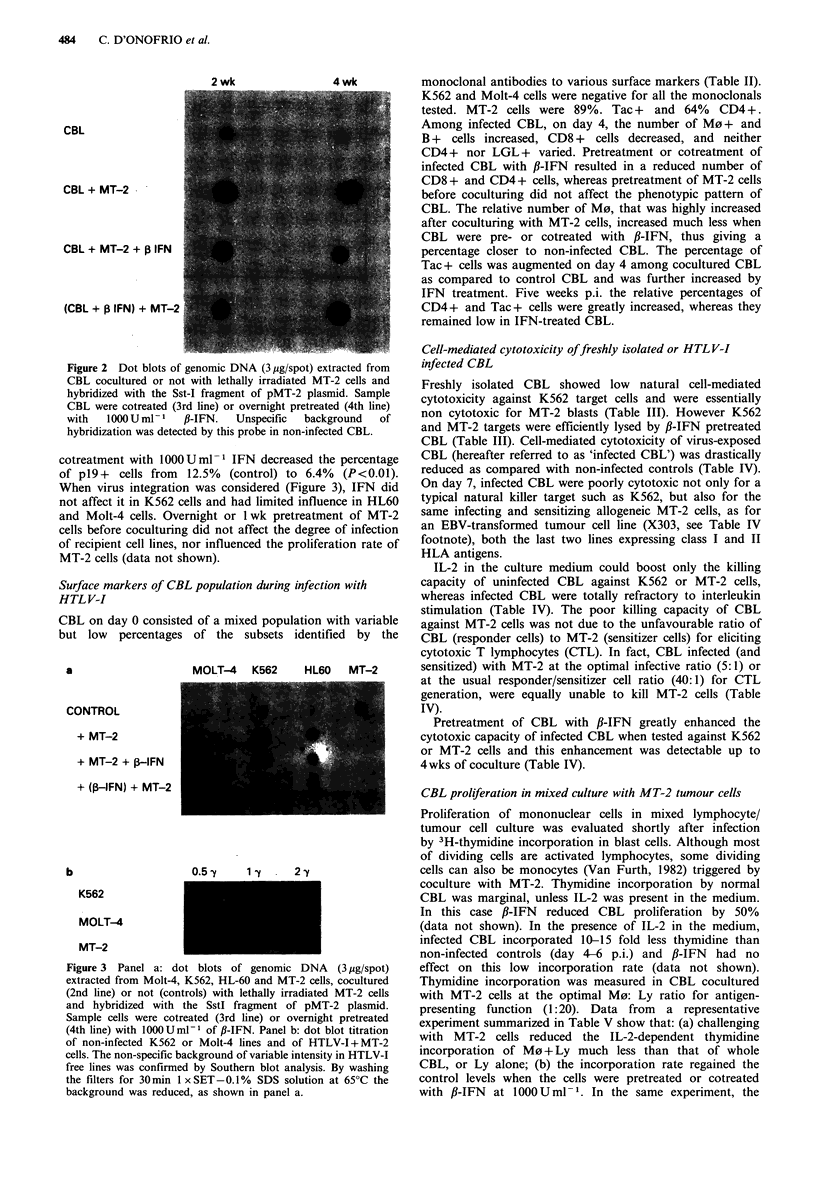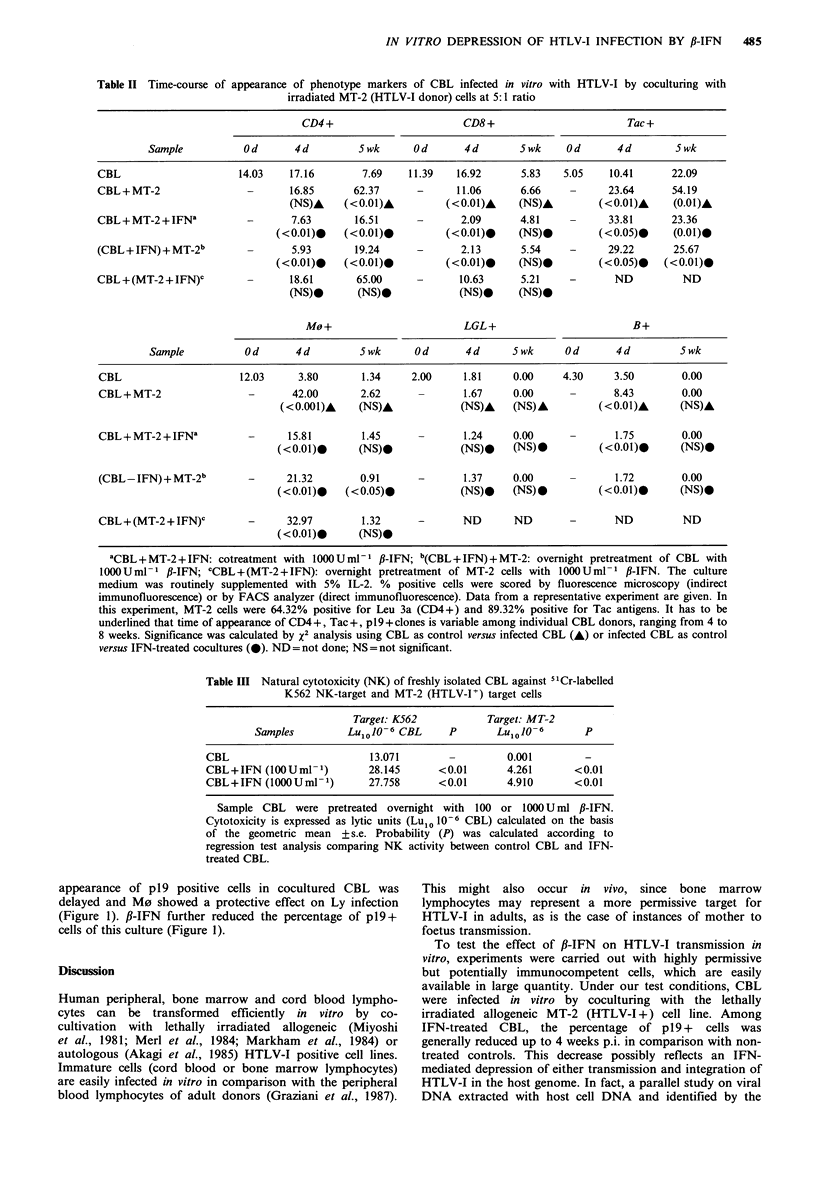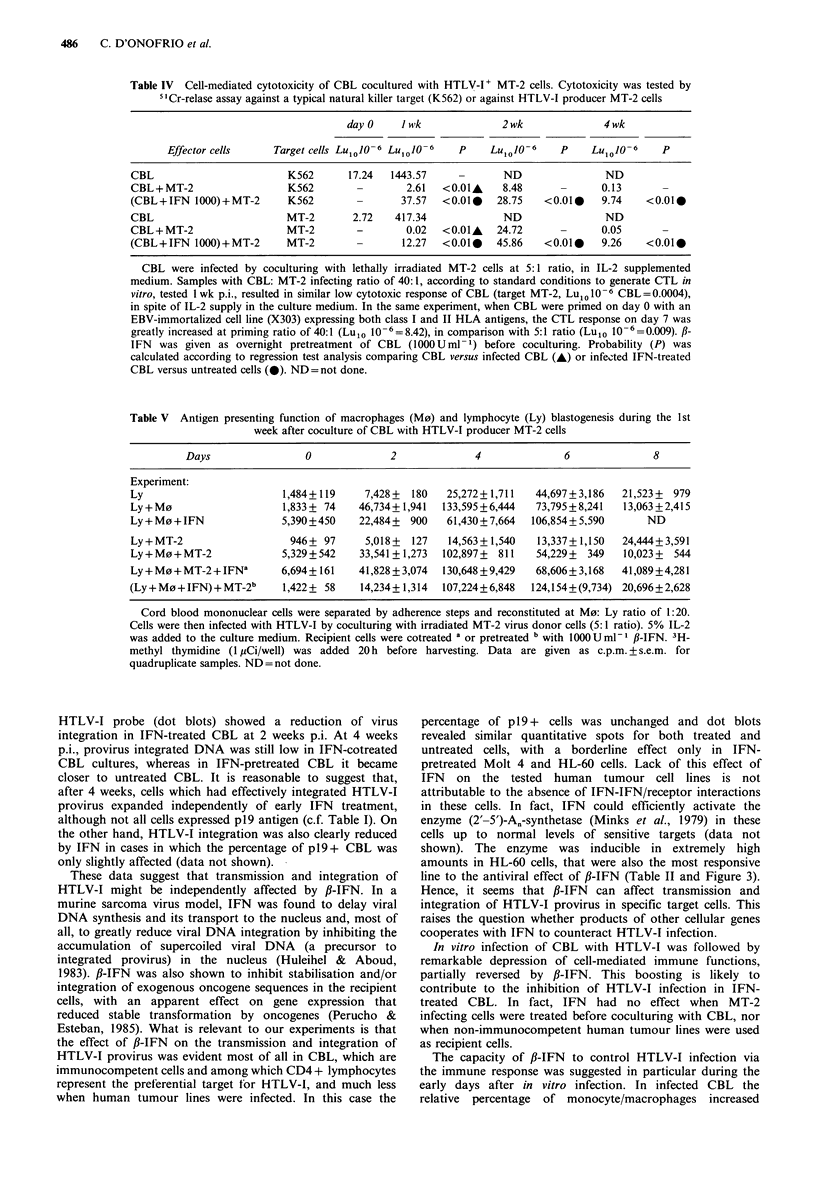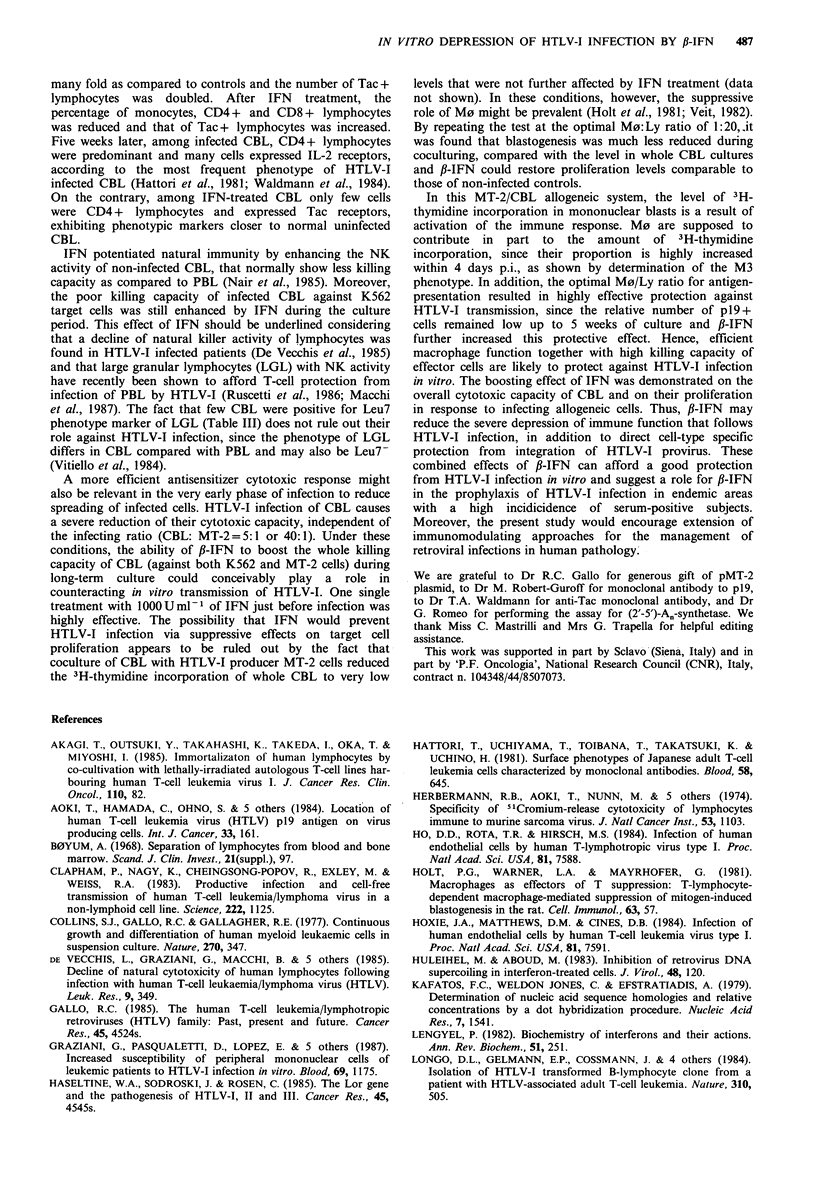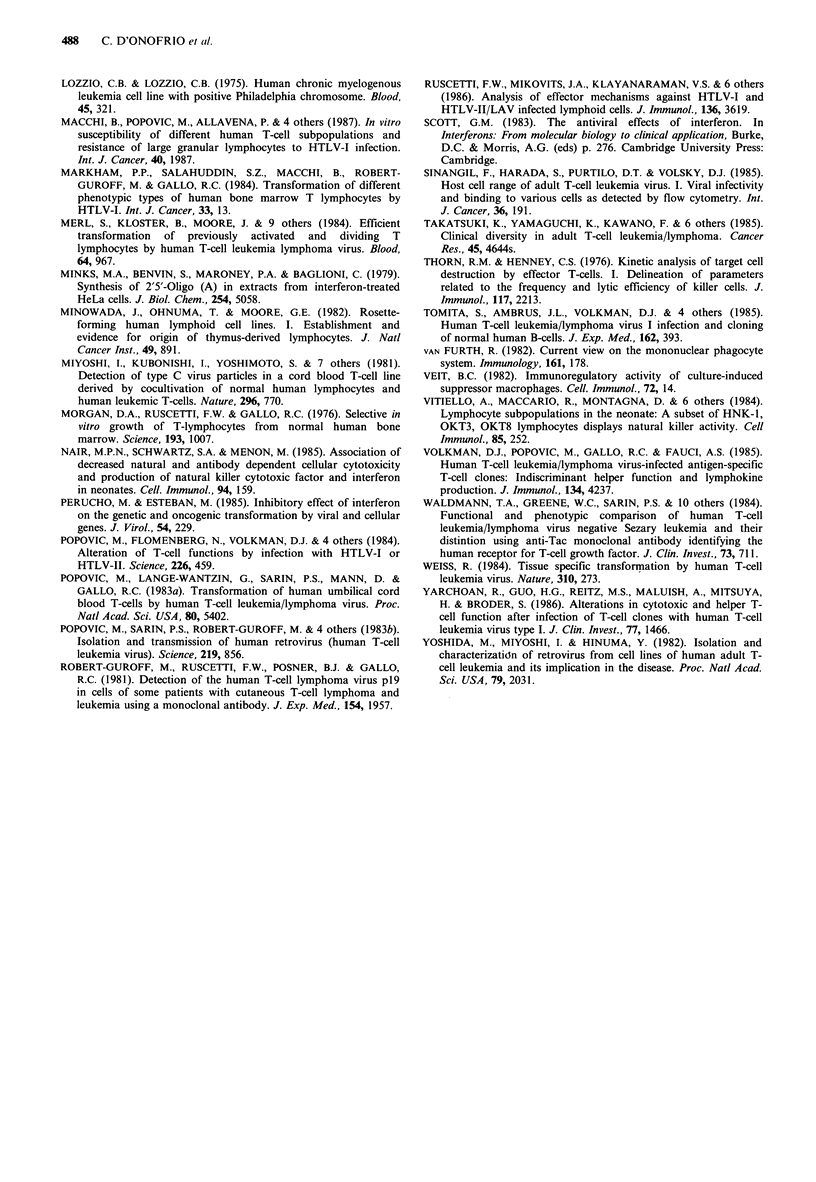# Depression of early phase of HTLV-I infection in vitro mediated by human beta-interferon.

**DOI:** 10.1038/bjc.1988.111

**Published:** 1988-05

**Authors:** C. D'Onofrio, C. F. Perno, P. Mazzetti, G. Graziani, R. Calio', E. Bonmassar

**Affiliations:** Department of Experimental Medicine and Biochemical Sciences, II University of Rome, Italy.

## Abstract

**Images:**


Br. J. Cancer (1988), 57, 481–488

© The Macmillan Press Ltd., 1988

### Depression of early phase of HTLV-I infection *in vitro* mediated by human beta-interferon

C. D'Onofrio, C.F. Perno, P. Mazzetti, G. Graziani, R. Calio' & E. Bonmassar

*Department of Experimental Medicine and Biochemical Sciences, II University of Rome, 00173 Rome, Italy.*

**Summary** Natural human interferon $\beta$ ($\beta$-IFN) was tested during the early phase of *in vitro* infection with HTLV-I virus of human cord blood mononuclear cells (CBL), to evaluate whether its antiviral and immunomodulating effects might prevent spreading of infection in the host. $\beta$-IFN was found to reduce HTLV-I transmission and integration in CBL cultures. Moreover, $\beta$-IFN had no effect in preventing virus transmission and integration in K562 and a very limited effect in HL60 and Molt-4 human tumour lines, suggesting a cell-type specific mode of action.

$\beta$-IFN induced a 'priming' response on CBL, since overnight pretreatment of recipient cells or one single treatment at the onset of the coculture were almost equally effective in protecting against HTLV-I infection. During the early days post infection (p.i.), IFN-treated CBL showed a pattern of phenotypic markers that was closer to that of non-infected CBL. In contrast, untreated CBL exposed to HTLV-I showed a percent increase of Tac+, M3+ and Leu 11+ subpopulations.

Cell-mediated immune responses of CBL were depressed after coculturing with HTLV-I producer MT-2 cells. $\beta$-IFN was able to boost the cell-mediated cytotoxicity of fresh and infected CBL against both K562 and MT-2 target cells. Leukocyte blastogenesis in mixed lymphocyte/tumour cell cultures, evaluated in terms of $^3$H-thymidine incorporation during the first week p.i., was also enhanced by IFN when macrophages and lymphocytes were reconstituted at an optimal 1:20 ratio. It is conceivable that this overall enhancement of the immune response induced by $\beta$-IFN could contribute to reduce HTLV-I infection *in vitro*.

Human T-cell leukaemia/lymphoma virus type I (HTLV-I), was the first human retrovirus to be associated with an aggressive form of adult T-cell leukaemia (ATL) (Gallo, 1985; Takatsuki *et al.*, 1985). HTLV-I is highly tropic for the CD4+ lymphocyte subset and integrates in the genome leading to transformation. The surface phenotype of ATL cells, as characterised by monoclonal antibodies, is CD3+, CD4+, CD8−, CD11− (Hattori *et al.*, 1981). ATL cells also express IL-2 receptors and class II HLA antigens (Waldmann *et al.*, 1984). However, HTLV-I transmission does not seem to be restricted to T lymphocytes, since B-cells (Longo *et al.*, 1984; Tomita *et al.*, 1985), some non-lymphoid cell lines (Clapham *et al.*, 1983) and primary endothelial cell cultures (Ho *et al.*, 1984; Hoxie *et al.*, 1984) can also be infected *in vitro*. Receptors for HTLV-I are indeed present on different types of cells from various species, although not all the receptor-positive cells are susceptible to stable infection (Sinangil *et al.*, 1985). The mechanisms of tissue-specific transformation mediated by HTLV-I are still unclear. It has been suggested that they are mediated by trans-acting transcriptional factor(s) of viral origin, that regulate(s) both HTLV promoter/enhancer sequences and activation of specific cellular genes controlling lymphocyte growth (Weiss, 1984; Haseltine *et al.*, 1985).

HTLV-I infection is accompanied by several alterations in immune function, including T-cell proliferation independently of IL-2 regulation (Popovic *et al.*, 1983*a,b*) and indiscriminant helper function (Popovic *et al.*, 1984; Volkam *et al.*, 1985; Yarchoan *et al.*, 1986). Since the specific target cell of HTLV-I is an immunocompetent cell, infection of T lymphocytes would result in a severe dysfunction of the immune system, which in turn could fail to control virus infection. Therefore, it is reasonable to hypothesize that immunosurveillance mechanisms play a significant role in controlling the early stages of HTLV-I transmission and preventing spreading of infection. In this respect, immunomodulating agents influence the rate and extent of target cell infection. They could protect from a massive horizontal transmission of HTLV-I and/or prevent the selection of the transformed clone(s).

Correspondence: C. D'Onofrio.
Received 6 April; and in revised form, 7 January 1988.

Interferons (IFNs) are potent antiviral and antitumour agents (Lengyel, 1982). $\alpha$ and $\beta$-IFN have been tested in clinical trials for therapy of virus-induced diseases (Scott, 1983). The effects of IFNs may be amplified by their known ability to modulate the immune system by regulating macrophage and lymphocyte functions. In view of this possibility, the effects of $\beta$-IFN on early stages of *in vitro* infection with HTLV-I have been tested. The present results show that $\beta$-IFN could effectively reduce both HTLV-I transmission and integration in human cord blood lymphocytes (CBL). This effect appeared to be cell type-specific and could be due, at least in part, to boosting of the immune function of recipient CBL.

### Materials and methods

### *Cell cultures and infection*

Human mononuclear cells were collected from heparinized neonatal umbilical cord blood (CBL) on Ficoll-Hypaque (Pharmacia, Uppsala, Sweden) gradients (1.077 g cm$^{-3}$) (Bøyum, 1968), seeded in 24-well plates or 25 cm$^2$ flasks (Falcon, Oxnard, USA) at a density of 0.5–1 $\times 10^6$ ml$^{-1}$ and incubated at 37°C in humidified air containing 5% $CO_2$. Human tumour lines were grown in 25 cm$^2$ flasks (Falcon) and diluted serially every 2 days. The culture medium was RPMI 1640 (Gibco, Grand Island, USA), plus 20% heat-inactivated foetal calf serum (FCS) (Gibco), 2 mM glutamine (Gibco) and 100 U ml$^{-1}$ penicillin/streptomycin. For CBL cultures, the medium was supplemented with 5% purified IL-2 (Cellular Products, Buffalo, USA) to guarantee the survival of lymphocytes (Morgan *et al.*, 1976). In long-term CBL cultures, the medium was renewed every week and fresh IL-2 added. Infection was achieved by coculturing CBL or recipient tumour lines with an HTLV-I producer line, MT-2 (Miyoshi *et al.*, 1981; Yoshida *et al.*, 1982), at a ratio of 5:1. MT-2 cells were lethally irradiated (120 Gy, Cesium Gamma Cell 1000, Canada Atomic Energy Ltd., Canada) before co-culturing. The human cell lines used as recipient cells were the erythroleukaemia K562 (Lozzio & Lozzio, 1975), the lymphoma Molt-4 (Minowada *et al.*, 1982) and the promyelocytic leukaemia HL-60 (Collins *et al.*, 1977) lines.

*Interferon treatment*

A freeze-dried preparation of natural β-IFN was kindly provided by Sclavo, Siena, Italy. It was resuspended in RPMI 1640 medium and aliquots were stored at −170°C. β-IFN was added to cell cultures at concentrations of 100 and $1000\,U\,ml^{-1}$. The scheme of treatments was as follows: (a) pretreatment of recipient cells overnight; (b) pretreatment of virus donor cells overnight; (c) treatment with β-IFN at the onset of the coculture (hereafter referred to as 'cotreatment'). After pretreatment according to scheme (a), recipient CBL were washed and transferred again in the same wells or flasks to keep unaltered the originally plated monocyte population.

*Indirect immunofluorescence for HTLV-I infected cells and surface phenotype of CBL*

To determine the percentage of HTLV-I infected cells, $\sim 5\times10^5$ cells were dispensed to 10 well multitest glass slides (Flow Laboratories, Irvine, UK), air-dried and fixed for 10 min in acetone/methanol (3:1); 10 μl 1:400 diluted monoclonal antibody to HTLV-I p19 core protein (Robert-Guroff *et al.*, 1981) were added to each spot for 30 min. After washing for 1 h in PBS plus 0.25% triton X100 (Sigma, St. Louis, USA), 10 μl 1:40 diluted rabbit anti-mouse IgG (Fab′ fragment) coupled with fluorescein-isothiocyanate (FITC) (Bio-Yeda, Rehovot, Israel) were added for 30 min. Samples were then washed overnight in PBS plus 0.25% triton X100, air-dried and mounted with glycerol. Percent positive cells were scored by fluorescence microscopy (Leitz, Wetzlar, FRG). MT-2 cells, nearly 100% $p19^+$, were taken as a positive control, and non-infected CBL or tumour cell lines as a negative control in each experiment.

The surface phenotype of CBL during *in vitro* infection with HTLV-I was determined by using monoclonal antibodies to surface markers. Leu 2a (CD8+), Leu 3a (CD4+), Leu 7 (LGL), Leu 12 (B lymphocytes) and M3 (monocytes/macrophages) were purchased from Becton-Dickinson (Sunnyvale, USA). Anti-Tac (IL-2 receptor) monoclonal antibody was kindly provided by T.A. Waldmann. Briefly, $10^6$ cells/vial were resuspended in 10 μl monoclonal antibody and incubated for 30 min on ice. After washing twice with PBS plus 5% FCS, cells were resuspended in 1:40 diluted rabbit anti mouse FITC-IgG or IgM (Bio-Yeda) for 30 min on ice, washed again, seeded on 10 well multitest glass slides (Flow) and air-dried. Samples were then fixed in ethanol/acetic acid (10:1) at −20°C, rehydrated by washing with cold PBS and mounted with glycerol and cover slips. Alternatively, the surface phenotype of CBL was evaluated by the direct immunofluorescence method (FITC-conjugated monoclonal antibodies, Becton-Dickinson) on a FACS analyzer (Becton-Dickinson).

*DNA extraction, DOT blots and hybridization procedure*

Genomic DNA was extracted from CBL or cell line pellets using the standard proteinase K method and resuspended in sterile TE buffer (Tris-HCl 10 mM, EDTA 1 mM). The yield was ~10 μg DNA $10^{-6}$ cells. RNA contamination of the samples was eliminated by digestion with DNAase-free RNAase. Dot blot analysis was performed according to Kafatos *et al.* (1979) as modified by G. Schütz. Briefly, samples were denatured (0.2 M NaOH for 10 min at 65°C), neutralized by adding 1 vol 2 M ammonium acetate, and spotted on nitrocellulose filter (Schleicher & Schüll, Dassel, FRG) previously saturated with 1 M ammonium acetate. Air-dried filters were baked for 2 h at 80°C. pMT-2 plasmid was kindly given by Dr R.C. Gallo; it contains the SstI-SstI fragment of HTLV-I (8.5 kb, accounting for almost the entire provirus sequences) cloned in pSP64. After digestion with SstI (Gibco/BRL, Eggenstein, FRG), the HTLV-I fragment was nick-translated by using $^{32}$P-dATP and $^{32}$P-dCTP nucleotides (Amersham Int., Amersham, UK). Specific activity of labelled probes was $\sim 1\times10^8\,cpm\,\mu g^{-1}$ DNA. Hybridization was performed in 1–5 ml of 10×Denhardt's solution-4×SET-0.1% SDS (20×SET = 3 M NaCl, 0.6 M Tris pH 8, 40 mM EDTA), in sealed plastic bags, at 65°C for 20 h. After hybridization, the filters were washed for 15 min in 10×Denhardt's-4×SET-0.1% SDS, 15 min in 2×SET-0.1% SDS at 65°C and exposed for 4–12 h at −80°C onto Kodak X AR-5 film (Kodak Company, Rochester, USA).

*Assay for cell mediated cytotoxicity*

Natural and antigen-specific cellular cytotoxicity of CBL were assayed from day 0 up to 4 weeks of coculture by $^{51}$Cr-release of labelled target cells (K562 or MT-2) in a 4 h test. Target cells were labelled in 0.1 ml FCS plus 100 μCi $^{51}$Cr-sodium chromate (Amersham) for 1 h at 37°C. Effector CBL ($2\times10^5$/well) were plated in round bottom 96-well microtiter plates. $^{51}$Cr-target cells were added at graded effector/target ratios, from 100:1 to 12.5:1. After 4 h incubation at 37°C in 5% $CO_2$ humidified air, 0.1 ml/well out of the 0.2 ml total supernatant was collected and counted in a gamma-scintillation counter (Packard, Instrument Co. Mod. A8000, Downers Grove, USA). Percent specific lysis was calculated according to the formula (Herberman *et al.*, 1974):

% specific lysis

$$= \frac{\text{cpm sample release-cpm autologous release}}{\text{total cpm}} \times 100$$

Autologous release represents the release of target cells incubated with non-labelled target cells as effector cells.

*Calculation of lytic units*

Dose-response curves were obtained by plotting the percentage of specific $^{51}$Cr release and the effector:target ratios. The best fit curve for this function was found to be logarithmic in accordance with previous reports (Thorn & Henney, 1976). A lytic unit n% (LUn) was defined as the number of effector cells, extrapolated from the dose-response curve, which were required to achieve n% specific target cell lysis. LUn $10^{-6}$ effector cells was calculated by dividing $10^6$ by the number of lymphocytes corresponding to 1 LUn.

*Assay for CBL proliferation in mixed lymphocyte/tumour culture*

CBL proliferation during coculture with MT-2 infecting cells was tested either in whole CBL cultures or at an optimal macrophage:lymphocyte ratio of 1:20, to enhance the antigen presenting function of macrophages (Mø). In this second case Mø were separated from lymphocytes (Ly) by a double adherence step, detached by a rubber policeman, and added to autologous non-adherent cells. β-IFN (100 or $1000\,U\,ml^{-1}$) was added as an overnight pretreatment of CBL or of MT-2 cells, or at the onset of the coculture. CBL blastogenesis was estimated on days 0, 2, 4, 6 and 8 by [methyl-$^3$H]-thymidine (2.6–3.1 TBeq $mmol^{-1}$, Amersham, UK) incorporation. $^3$H-thymidine was used at the concentration of 1 μCi per $2\times10^5$ cells per well. Samples were harvested 20 h later by microtiter cell harvester (Titertek 530, Flow Laboratories) and counted in a scintillation beta-counter (LKB, Bromma, Sweden).

### Results

In vitro *infection of CBL with HTLV-I*

*In vitro* infection of CBL with HTLV-I was obtained by using an optimal CBL: HTLV-I donor-cell ratio (5:1) and supplementing the culture medium with IL-2, to allow the survival of lymphocytes. Under these standard conditions, CBL were susceptible to HTLV-I infection to a variable extent depending in part on different cord blood donors. CBL generally responded to challenge with virus-infected allogeneic cells with a modest initial proliferation that decreased within 1–2 weeks, and cells passed through a growth crisis at ~3–4 weeks, then the transformed clone(s) started to expand (data not shown). Addition of β-IFN to CBL/MT-2 cocultures (i.e., cotreatment with β-IFN) did not affect the low proliferative response of CBL (data not shown). Similarly β-IFN did not depress the proliferation rate of MT-2 cocultures with K562, HL60 or Molt-4 cells. Moreover, continuous treatment of MT-2 or K562 cells with β-IFN (1000 U $ml^{-1}$ every 2 days) for 1 week did not inhibit cell growth (data not shown).

HTLV-I transmission was evaluated by indirect immunofluorescence for the p19 virus core protein in 10 CBL/MT-2 cocultures. A typical experiment, illustrated in Table I, shows the infection pattern of most CBL cultures: (a) few cells were positive for the p19 protein until 2–3 weeks of culture; (b) after 4 weeks this percentage increased in untreated CBL, but remained very low in IFN-treated CBL; (c) similar results were obtained both when CBL were pretreated with IFN before coculturing and when IFN was added at the onset of the coculture; (d) pretreatment of MT-2 cells before irradiation and coculture had no effect on the percentage of p19+ cells as compared to controls. When CBL were fractionated and reconstituted to optimal Mø:Ly ratio (1:20) for antigen-presenting function, the presence of Mø delayed the appearance of p19+ cells when compared to Ly alone (Figure 1). However, IFN afforded further protection against HTLV-I, as evidenced by the significantly lower number of p19+ cells in treated Mø+Ly cultures as compared to untreated controls, 5 weeks p.i. (Figure 1). Positivity for the p19 protein indicates the presence of virions on the membrane or inside the cells (Aoki *et al.*, 1984), but does not directly correlate to virus integration in the host genome. By this test one cannot discriminate between virus uptake and integration in infected cells. Hence, dot blot analysis of genomic DNA of CBL, hybridized with the Sst-I fragment as probe, was performed to verify whether p19 expression correlated with virus integration. As shown in Figure 2, the integration of HTLV-I in 2 wk cocultured CBL was quantitatively reduced after IFN treatment. IFN was effective when used for pretreating CBL, and also when given as cotreatment. In a 4 wk culture, a clear difference was still observed among IFN-cotreated CBL in comparison with untreated CBL. On the contrary, IFN-pretreated CBL showed a spot quantitatively similar to untreated cells. In one out of 5 whole CBL cocultures,

**Figure 1** Time-course of p19 positivity of CBL-derived Ly (○——○) or Mø+Ly (●——●) (1:20) cocultured with lethally irradiated MT-2 (HTLV-I donor) cells at 5:1 ratio. β-IFN-pretreated (▲——▲) or cotreated (■——■) (1000 U $ml^{-1}$) Ly+Mø cultures were significantly less positive for p19 than untreated Ly+Mø cultures ($P<0.01$ at 5 weeks). Mø exerted a protective effect on Ly during coculture ($P<0.01$ at 4 weeks and $P<0.05$ at 5 weeks comparing Ly to Ly+Mø cultures). Probability was calculated according to $\chi^2$ analysis.

however, the number of p19+ cells was relatively high (20–25%) 2–3 weeks p.i. and IFN had slight effect, if any, in decreasing this percentage. Even in this case, however, provirus integration was greatly impaired by IFN pretreatment or cotreatment, as indicated by dot blot analysis performed 2 weeks p.i. (data not shown). This observation indicates that HTLV-I transmission and integration can also be independently affected by β-IFN.

### In vitro *infection of human tumour cells line*

K562, Molt-4 and HL-60 cells were infected by coculturing with MT-2 cells as for CBL cultures. Viral p19 was assayed by indirect immunofluorescence 5 days p.i., since HTLV-I was cytopathic for recipient cells and their viability on day 5 was generally reduced to 40–50%, approaching total cell death after 10 days.

All 3 cell lines were susceptible to HTLV-I infection and 10–20% cells became p19+ after 5 days (data not shown). Pretreatment of the recipient line or cotreatment with β-IFN had no effect on HTLV-I infection, with the exception of one out of two experiments with HL60, in which

**Table I** Time-course of appearance of p19 positive cells among CBL infected *in vitro* with HTLV-I

| | *1 wk* | | *2 wk* | | *4 wk* | |
|---|---|---|---|---|---|---|
| *Samples* | *% p19* | *P* | *% p19* | *P* | *% p19* | *P* |
| CBL+MT-2 (controls) | 7.62 | – | 13.07 | – | 31.47 | – |
| CBL+MT-2+IFN 100[a] | 6.85 | NS | 5.81 | <0.01 | 17.93 | <0.01 |
| CBL+MT-2+IFN 1000[a] | 6.96 | NS | 3.46 | <0.01 | 9.95 | <0.01 |
| (CBL IFN 100)+MT-2[b] | 8.19 | NS | 4.00 | <0.01 | 16.97 | <0.01 |
| (CBL IFN 1000)+MT-2[b] | 7.80 | NS | 5.29 | <0.01 | 12.08 | <0.01 |
| CBL+(MT-2 IFN 100)[c] | 6.04 | NS | ND | | 26.79 | NS |
| CBL+(MT-2 IFN 1000)[c] | 4.83 | NS | ND | | 31.95 | NS |

Scheme of treatments: [a]Cotreatment of CBL with 100 or 1000 U $ml^{-1}$ β-IFN; [b](CBL IFN): overnight pretreatment of CBL with 100 or 1000 U $ml^{-1}$ β-IFN; [c](MT-2 IFN): overnight pretreatment of MT-2 cells (HTLV-I donor cells) with 100 or 1000 U $ml^{-1}$. N.D. = not done. CBL were then cocultured with irradiated MT-2 cells at 5:1 ratio. Infection was evaluated by indirect immunofluorescence for the p19 virus core protein. Significance (*P*) was calculated by $\chi^2$ analysis comparing % p19 between CBL+MT-2 and IFN-treated samples. NS: not significant. Per cent inhibition of p19 positivity 2 wks p.i. in 10 CBL/MT-2 cocultures ranged from 13% to 85%.

**Figure 2** Dot blots of genomic DNA (3 μg/spot) extracted from CBL cocultured or not with lethally irradiated MT-2 cells and hybridized with the Sst-I fragment of pMT-2 plasmid. Sample CBL were cotreated (3rd line) or overnight pretreated (4th line) with 1000 U ml$^{-1}$ β-IFN. Unspecific background of hybridization was detected by this probe in non-infected CBL.

cotreatment with 1000 U ml$^{-1}$ IFN decreased the percentage of p19+ cells from 12.5% (control) to 6.4% ($P<0.01$). When virus integration was considered (Figure 3), IFN did not affect it in K562 cells and had limited influence in HL60 and Molt-4 cells. Overnight or 1 wk pretreatment of MT-2 cells before coculturing did not affect the degree of infection of recipient cell lines, nor influenced the proliferation rate of MT-2 cells (data not shown).

*Surface markers of CBL population during infection with HTLV-I*

CBL on day 0 consisted of a mixed population with variable but low percentages of the subsets identified by the

**Figure 3** Panel a: dot blots of genomic DNA (3 μg/spot) extracted from Molt-4, K562, HL-60 and MT-2 cells, cocultured (2nd line) or not (controls) with lethally irradiated MT-2 cells and hybridized with the SstI fragment of pMT-2 plasmid. Sample cells were cotreated (3rd line) or overnight pretreated (4th line) with 1000 U ml$^{-1}$ of β-IFN. Panel b: dot blot titration of non-infected K562 or Molt-4 lines and of HTLV-I+MT-2 cells. The non-specific background of variable intensity in HTLV-I free lines was confirmed by Southern blot analysis. By washing the filters for 30 min 1×SET−0.1% SDS solution at 65°C the background was reduced, as shown in panel a.

monoclonal antibodies to various surface markers (Table II). K562 and Molt-4 cells were negative for all the monoclonals tested. MT-2 cells were 89%. Tac+ and 64% CD4+. Among infected CBL, on day 4, the number of Mø+ and B+ cells increased, CD8+ cells decreased, and neither CD4+ nor LGL+ varied. Pretreatment or cotreatment of infected CBL with β-IFN resulted in a reduced number of CD8+ and CD4+ cells, whereas pretreatment of MT-2 cells before coculturing did not affect the phenotypic pattern of CBL. The relative number of Mø, that was highly increased after coculturing with MT-2 cells, increased much less when CBL were pre- or cotreated with β-IFN, thus giving a percentage closer to non-infected CBL. The percentage of Tac+ cells was augmented on day 4 among cocultured CBL as compared to control CBL and was further increased by IFN treatment. Five weeks p.i. the relative percentages of CD4+ and Tac+ cells were greatly increased, whereas they remained low in IFN-treated CBL.

*Cell-mediated cytotoxicity of freshly isolated or HTLV-I infected CBL*

Freshly isolated CBL showed low natural cell-mediated cytotoxicity against K562 target cells and were essentially non cytotoxic for MT-2 blasts (Table III). However K562 and MT-2 targets were efficiently lysed by β-IFN pretreated CBL (Table III). Cell-mediated cytotoxicity of virus-exposed CBL (hereafter referred to as 'infected CBL') was drastically reduced as compared with non-infected controls (Table IV). On day 7, infected CBL were poorly cytotoxic not only for a typical natural killer target such as K562, but also for the same infecting and sensitizing allogeneic MT-2 cells, as for an EBV-transformed tumour cell line (X303, see Table IV footnote), both the last two lines expressing class I and II HLA antigens.

IL-2 in the culture medium could boost only the killing capacity of uninfected CBL against K562 or MT-2 cells, whereas infected CBL were totally refractory to interleukin stimulation (Table IV). The poor killing capacity of CBL against MT-2 cells was not due to the unfavourable ratio of CBL (responder cells) to MT-2 (sensitizer cells) for eliciting cytotoxic T lymphocytes (CTL). In fact, CBL infected (and sensitized) with MT-2 at the optimal infective ratio (5:1) or at the usual responder/sensitizer cell ratio (40:1) for CTL generation, were equally unable to kill MT-2 cells (Table IV).

Pretreatment of CBL with β-IFN greatly enhanced the cytotoxic capacity of infected CBL when tested against K562 or MT-2 cells and this enhancement was detectable up to 4 wks of coculture (Table IV).

*CBL proliferation in mixed culture with MT-2 tumour cells*

Proliferation of mononuclear cells in mixed lymphocyte/tumour cell culture was evaluated shortly after infection by $^{3}$H-thymidine incorporation in blast cells. Although most of dividing cells are activated lymphocytes, some dividing cells can also be monocytes (Van Furth, 1982) triggered by coculture with MT-2. Thymidine incorporation by normal CBL was marginal, unless IL-2 was present in the medium. In this case β-IFN reduced CBL proliferation by 50% (data not shown). In the presence of IL-2 in the medium, infected CBL incorporated 10–15 fold less thymidine than non-infected controls (day 4–6 p.i.) and β-IFN had no effect on this low incorporation rate (data not shown). Thymidine incorporation was measured in CBL cocultured with MT-2 cells at the optimal Mø: Ly ratio for antigen-presenting function (1:20). Data from a representative experiment summarized in Table V show that: (a) challenging with MT-2 cells reduced the IL-2-dependent thymidine incorporation of Mø+Ly much less than that of whole CBL, or Ly alone; (b) the incorporation rate regained the control levels when the cells were pretreated or cotreated with β-IFN at 1000 U ml$^{-1}$. In the same experiment, the

**Table II** Time-course of appearance of phenotype markers of CBL infected *in vitro* with HTLV-I by coculturing with irradiated MT-2 (HTLV-I donor) cells at 5:1 ratio

| | *CD4+* | | | *CD8+* | | | *Tac+* | | |
|---|---|---|---|---|---|---|---|---|---|
| *Sample* | *0 d* | *4 d* | *5 wk* | *0 d* | *4 d* | *5 wk* | *0 d* | *4 d* | *5 wk* |
| CBL | 14.03 | 17.16 | 7.69 | 11.39 | 16.92 | 5.83 | 5.05 | 10.41 | 22.09 |
| CBL+MT-2 | – | 16.85 (NS)▲ | 62.37 (<0.01)▲ | – | 11.06 (<0.01)▲ | 6.66 (NS)▲ | – | 23.64 (<0.01)▲ | 54.19 (0.01)▲ |
| CBL+MT-2+IFN[a] | – | 7.63 (<0.01)● | 16.51 (<0.01)● | – | 2.09 (<0.01)● | 4.81 (NS)● | – | 33.81 (<0.05)● | 23.36 (0.01)● |
| (CBL+IFN)+MT-2[b] | – | 5.93 (<0.01)● | 19.24 (<0.01)● | – | 2.13 (<0.01)● | 5.54 (NS)● | – | 29.22 (<0.05)● | 25.67 (<0.01)● |
| CBL+(MT-2+IFN)[c] | – | 18.61 (NS)● | 65.00 (NS)● | – | 10.63 (NS)● | 5.21 (NS)● | – | ND | ND |
| | *Mø+* | | | *LGL+* | | | *B+* | | |
| *Sample* | *0 d* | *4 d* | *5 wk* | *0 d* | *4 d* | *5 wk* | *0 d* | *4 d* | *5 wk* |
| CBL | 12.03 | 3.80 | 1.34 | 2.00 | 1.81 | 0.00 | 4.30 | 3.50 | 0.00 |
| CBL+MT-2 | – | 42.00 (<0.001)▲ | 2.62 (NS)▲ | – | 1.67 (NS)▲ | 0.00 (NS)▲ | – | 8.43 (<0.01)▲ | 0.00 (NS)▲ |
| CBL+MT-2+IFN[a] | – | 15.81 (<0.01)● | 1.45 (NS)● | – | 1.24 (NS)● | 0.00 (NS)● | – | 1.75 (<0.01)● | 0.00 (NS)● |
| (CBL−IFN)+MT-2[b] | – | 21.32 (<0.01)● | 0.91 (<0.05)● | – | 1.37 (NS)● | 0.00 (NS)● | – | 1.72 (<0.01)● | 0.00 (NS)● |
| CBL+(MT-2+IFN)[c] | – | 32.97 (<0.01)● | 1.32 (NS)● | – | ND | ND | – | ND | ND |

[a]CBL+MT-2+IFN: cotreatment with 1000 U ml$^{-1}$ β-IFN; [b](CBL+IFN)+MT-2: overnight pretreatment of CBL with 1000 U ml$^{-1}$ β-IFN; [c]CBL+(MT-2+IFN): overnight pretreatment of MT-2 cells with 1000 U ml$^{-1}$ β-IFN. The culture medium was routinely supplemented with 5% IL-2. % positive cells were scored by fluorescence microscopy (indirect immunofluorescence) or by FACS analyzer (direct immunofluorescence). Data from a representative experiment are given. In this experiment, MT-2 cells were 64.32% positive for Leu 3a (CD4+) and 89.32% positive for Tac antigens. It has to be underlined that time of appearance of CD4+, Tac+, p19+clones is variable among individual CBL donors, ranging from 4 to 8 weeks. Significance was calculated by $\chi^2$ analysis using CBL as control *versus* infected CBL (▲) or infected CBL as control *versus* IFN-treated cocultures (●). ND = not done; NS = not significant.

**Table III** Natural cytotoxicity (NK) of freshly isolated CBL against $^{51}$Cr-labelled K562 NK-target and MT-2 (HTLV-I$^{+}$) target cells

| *Samples* | *Target: K562* $Lu_{10} 10^{-6}$ *CBL* | *P* | *Target: MT-2* $Lu_{10} 10^{-6}$ | *P* |
|---|---|---|---|---|
| CBL | 13.071 | – | 0.001 | – |
| CBL+IFN (100 U ml$^{-1}$) | 28.145 | <0.01 | 4.261 | <0.01 |
| CBL+IFN (1000 U ml$^{-1}$) | 27.758 | <0.01 | 4.910 | <0.01 |

Sample CBL were pretreated overnight with 100 or 1000 U ml β-IFN. Cytotoxicity is expressed as lytic units ($Lu_{10}$ $10^{-6}$ CBL) calculated on the basis of the geometric mean ±s.e. Probability (*P*) was calculated according to regression test analysis comparing NK activity between control CBL and IFN-treated CBL.

appearance of p19 positive cells in cocultured CBL was delayed and Mø showed a protective effect on Ly infection (Figure 1). β-IFN further reduced the percentage of p19+ cells of this culture (Figure 1).

### Discussion

Human peripheral, bone marrow and cord blood lymphocytes can be transformed efficiently *in vitro* by co-cultivation with lethally irradiated allogeneic (Miyoshi *et al.*, 1981; Merl *et al.*, 1984; Markham *et al.*, 1984) or autologous (Akagi *et al.*, 1985) HTLV-I positive cell lines. Immature cells (cord blood or bone marrow lymphocytes) are easily infected *in vitro* in comparison with the peripheral blood lymphocytes of adult donors (Graziani *et al.*, 1987). This might also occur *in vivo*, since bone marrow lymphocytes may represent a more permissive target for HTLV-I in adults, as is the case of instances of mother to foetus transmission.

To test the effect of β-IFN on HTLV-I transmission *in vitro*, experiments were carried out with highly permissive but potentially immunocompetent cells, which are easily available in large quantity. Under our test conditions, CBL were infected *in vitro* by coculturing with the lethally irradiated allogeneic MT-2 (HTLV-I+) cell line. Among IFN-treated CBL, the percentage of p19+ cells was generally reduced up to 4 weeks p.i. in comparison with non-treated controls. This decrease possibly reflects an IFN-mediated depression of either transmission and integration of HTLV-I in the host genome. In fact, a parallel study on viral DNA extracted with host cell DNA and identified by the

**Table IV** Cell-mediated cytotoxicity of CBL cocultured with HTLV-I+ MT-2 cells. Cytotoxicity was tested by $^{51}$Cr-relase assay against a typical natural killer target (K562) or against HTLV-I producer MT-2 cells

| | | day 0 | 1 wk | | 2 wk | | 4 wk | |
|---|---|---|---|---|---|---|---|---|
| *Effector cells* | *Target cells* | *$Lu_{10}10^{-6}$* | *$Lu_{10}10^{-6}$* | *P* | *$Lu_{10}10^{-6}$* | *P* | *$Lu_{10}10^{-6}$* | *P* |
| CBL | K562 | 17.24 | 1443.57 | – | ND | | ND | |
| CBL+MT-2 | K562 | – | 2.61 | <0.01▲ | 8.48 | – | 0.13 | – |
| (CBL+IFN 1000)+MT-2 | K562 | – | 37.57 | <0.01● | 28.75 | <0.01● | 9.74 | <0.01● |
| CBL | MT-2 | 2.72 | 417.34 | | ND | | ND | |
| CBL+MT-2 | MT-2 | – | 0.02 | <0.01▲ | 24.72 | – | 0.05 | – |
| (CBL+IFN 1000)+MT-2 | MT-2 | – | 12.27 | <0.01● | 45.86 | <0.01● | 9.26 | <0.01● |

CBL were infected by coculturing with lethally irradiated MT-2 cells at 5:1 ratio, in IL-2 supplemented medium. Samples with CBL: MT-2 infecting ratio of 40:1, according to standard conditions to generate CTL *in vitro*, tested 1 wk p.i., resulted in similar low cytotoxic response of CBL (target MT-2, $Lu_{10}10^{-6}$ CBL=0.0004), in spite of IL-2 supply in the culture medium. In the same experiment, when CBL were primed on day 0 with an EBV-immortalized cell line (X303) expressing both class I and II HLA antigens, the CTL response on day 7 was greatly increased at priming ratio of 40:1 ($Lu_{10}$ $10^{-6}$=8.42), in comparison with 5:1 ratio ($Lu_{10}$ $10^{-6}$=0.009). β-IFN was given as overnight pretreatment of CBL (1000 U ml$^{-1}$) before coculturing. Probability (*P*) was calculated according to regression test analysis comparing CBL *versus* infected CBL (▲) or infected IFN-treated CBL versus untreated cells (●). ND=not done.

**Table V** Antigen presenting function of macrophages (Mø) and lymphocyte (Ly) blastogenesis during the 1st week after coculture of CBL with HTLV-I producer MT-2 cells

| *Days* | *0* | *2* | *4* | *6* | *8* |
|---|---|---|---|---|---|
| Experiment: | | | | | |
| Ly | 1,484±119 | 7,428± 180 | 25,272±1,711 | 44,697±3,186 | 21,523± 979 |
| Ly+Mø | 1,833± 74 | 46,734±1,941 | 133,595±6,444 | 73,795±8,241 | 13,063±2,415 |
| Ly+Mø+IFN | 5,390±450 | 22,484± 900 | 61,430±7,664 | 106,854±5,590 | ND |
| Ly+MT-2 | 946± 97 | 5,018± 127 | 14,563±1,540 | 13,337±1,150 | 24,444±3,591 |
| Ly+Mø+MT-2 | 5,329±542 | 33,541±1,273 | 102,897± 811 | 54,229± 349 | 10,023± 544 |
| Ly+Mø+MT-2+IFN[a] | 6,694±161 | 41,828±3,074 | 130,648±9,429 | 68,606±3,168 | 41,089±4,281 |
| (Ly+Mø+IFN)+MT-2[b] | 1,422± 58 | 14,234±1,314 | 107,224±6,848 | 124,154±(9,734) | 20,696±2,628 |

Cord blood mononuclear cells were separated by adherence steps and reconstituted at Mø: Ly ratio of 1:20. Cells were then infected with HTLV-I by coculturing with irradiated MT-2 virus donor cells (5:1 ratio). 5% IL-2 was added to the culture medium. Recipient cells were cotreated [a] or pretreated [b] with 1000 U ml$^{-1}$ β-IFN. $^{3}$H-methyl thymidine (1 μCi/well) was added 20 h before harvesting. Data are given as c.p.m.±s.e.m. for quadruplicate samples. ND=not done.

HTLV-I probe (dot blots) showed a reduction of virus integration in IFN-treated CBL at 2 weeks p.i. At 4 weeks p.i., provirus integrated DNA was still low in IFN-cotreated CBL cultures, whereas in IFN-pretreated CBL it became closer to untreated CBL. It is reasonable to suggest that, after 4 weeks, cells which had effectively integrated HTLV-I provirus expanded independently of early IFN treatment, although not all cells expressed p19 antigen (c.f. Table I). On the other hand, HTLV-I integration was also clearly reduced by IFN in cases in which the percentage of p19+ CBL was only slightly affected (data not shown).

These data suggest that transmission and integration of HTLV-I might be independently affected by β-IFN. In a murine sarcoma virus model, IFN was found to delay viral DNA synthesis and its transport to the nucleus and, most of all, to greatly reduce viral DNA integration by inhibiting the accumulation of supercoiled viral DNA (a precursor to integrated provirus) in the nucleus (Huleihel & Aboud, 1983). β-IFN was also shown to inhibit stabilisation and/or integration of exogenous oncogene sequences in the recipient cells, with an apparent effect on gene expression that reduced stable transformation by oncogenes (Perucho & Esteban, 1985). What is relevant to our experiments is that the effect of β-IFN on the transmission and integration of HTLV-I provirus was evident most of all in CBL, which are immunocompetent cells and among which CD4+ lymphocytes represent the preferential target for HTLV-I, and much less when human tumour lines were infected. In this case the percentage of p19+ cells was unchanged and dot blots revealed similar quantitative spots for both treated and untreated cells, with a borderline effect only in IFN-pretreated Molt 4 and HL-60 cells. Lack of this effect of IFN on the tested human tumour cell lines is not attributable to the absence of IFN-IFN/receptor interactions in these cells. In fact, IFN could efficiently activate the enzyme (2′–5′)-$A_n$-synthetase (Minks *et al.*, 1979) in these cells up to normal levels of sensitive targets (data not shown). The enzyme was inducible in extremely high amounts in HL-60 cells, that were also the most responsive line to the antiviral effect of β-IFN (Table II and Figure 3). Hence, it seems that β-IFN can affect transmission and integration of HTLV-I provirus in specific target cells. This raises the question whether products of other cellular genes cooperates with IFN to counteract HTLV-I infection.

*In vitro* infection of CBL with HTLV-I was followed by remarkable depression of cell-mediated immune functions, partially reversed by β-IFN. This boosting is likely to contribute to the inhibition of HTLV-I infection in IFN-treated CBL. In fact, IFN had no effect when MT-2 infecting cells were treated before coculturing with CBL, nor when non-immunocompetent human tumour lines were used as recipient cells.

The capacity of β-IFN to control HTLV-I infection via the immune response was suggested in particular during the early days after *in vitro* infection. In infected CBL the relative percentage of monocyte/macrophages increased many fold as compared to controls and the number of Tac+ lymphocytes was doubled. After IFN treatment, the percentage of monocytes, CD4+ and CD8+ lymphocytes was reduced and that of Tac+ lymphocytes was increased. Five weeks later, among infected CBL, CD4+ lymphocytes were predominant and many cells expressed IL-2 receptors, according to the most frequent phenotype of HTLV-I infected CBL (Hattori *et al.*, 1981; Waldmann *et al.*, 1984). On the contrary, among IFN-treated CBL only few cells were CD4+ lymphocytes and expressed Tac receptors, exhibiting phenotypic markers closer to normal uninfected CBL.

IFN potentiated natural immunity by enhancing the NK activity of non-infected CBL, that normally show less killing capacity as compared to PBL (Nair *et al.*, 1985). Moreover, the poor killing capacity of infected CBL against K562 target cells was still enhanced by IFN during the culture period. This effect of IFN should be underlined considering that a decline of natural killer activity of lymphocytes was found in HTLV-I infected patients (De Vecchis *et al.*, 1985) and that large granular lymphocytes (LGL) with NK activity have recently been shown to afford T-cell protection from infection of PBL by HTLV-I (Ruscetti *et al.*, 1986; Macchi *et al.*, 1987). The fact that few CBL were positive for Leu7 phenotype marker of LGL (Table III) does not rule out their role against HTLV-I infection, since the phenotype of LGL differs in CBL compared with PBL and may also be $Leu7^-$ (Vitiello *et al.*, 1984).

A more efficient antisensitizer cytotoxic response might also be relevant in the very early phase of infection to reduce spreading of infected cells. HTLV-I infection of CBL causes a severe reduction of their cytotoxic capacity, independent of the infecting ratio (CBL: MT-2 = 5:1 or 40:1). Under these conditions, the ability of β-IFN to boost the whole killing capacity of CBL (against both K562 and MT-2 cells) during long-term culture could conceivably play a role in counteracting *in vitro* transmission of HTLV-I. One single treatment with $1000\,U\,ml^{-1}$ of IFN just before infection was highly effective. The possibility that IFN would prevent HTLV-I infection via suppressive effects on target cell proliferation appears to be ruled out by the fact that coculture of CBL with HTLV-I producer MT-2 cells reduced the $^3$H-thymidine incorporation of whole CBL to very low levels that were not further affected by IFN treatment (data not shown). In these conditions, however, the suppressive role of Mø might be prevalent (Holt *et al.*, 1981; Veit, 1982). By repeating the test at the optimal Mø:Ly ratio of 1:20, it was found that blastogenesis was much less reduced during coculturing, compared with the level in whole CBL cultures and β-IFN could restore proliferation levels comparable to those of non-infected controls.

In this MT-2/CBL allogeneic system, the level of $^3$H-thymidine incorporation in mononuclear blasts is a result of activation of the immune response. Mø are supposed to contribute in part to the amount of $^3$H-thymidine incorporation, since their proportion is highly increased within 4 days p.i., as shown by determination of the M3 phenotype. In addition, the optimal Mø/Ly ratio for antigen-presentation resulted in highly effective protection against HTLV-I transmission, since the relative number of p19+ cells remained low up to 5 weeks of culture and β-IFN further increased this protective effect. Hence, efficient macrophage function together with high killing capacity of effector cells are likely to protect against HTLV-I infection *in vitro*. The boosting effect of IFN was demonstrated on the overall cytotoxic capacity of CBL and on their proliferation in response to infecting allogeneic cells. Thus, β-IFN may reduce the severe depression of immune function that follows HTLV-I infection, in addition to direct cell-type specific protection from integration of HTLV-I provirus. These combined effects of β-IFN can afford a good protection from HTLV-I infection *in vitro* and suggest a role for β-IFN in the prophylaxis of HTLV-I infection in endemic areas with a high incidicidence of serum-positive subjects. Moreover, the present study would encourage extension of immunomodulating approaches for the management of retroviral infections in human pathology.

We are grateful to Dr R.C. Gallo for generous gift of pMT-2 plasmid, to Dr M. Robert-Guroff for monoclonal antibody to p19, to Dr T.A. Waldmann for anti-Tac monoclonal antibody, and Dr G. Romeo for performing the assay for (2′-5′)-$A_n$-synthetase. We thank Miss C. Mastrilli and Mrs G. Trapella for helpful editing assistance.

This work was supported in part by Sclavo (Siena, Italy) and in part by 'P.F. Oncologia', National Research Council (CNR), Italy, contract n. 104348/44/8507073.

### References

AKAGI, T., OUTSUKI, Y., TAKAHASHI, K., TAKEDA, I., OKA, T. & MIYOSHI, I. (1985). Immortalizaton of human lymphocytes by co-cultivation with lethally-irradiated autologous T-cell lines harbouring human T-cell leukemia virus I. *J. Cancer Res. Clin. Oncol.*, **110**, 82.

AOKI, T., HAMADA, C., OHNO, S. & 5 others (1984). Location of human T-cell leukemia virus (HTLV) p19 antigen on virus producing cells. *Int. J. Cancer*, **33**, 161.

BØYUM, A. (1968). Separation of lymphocytes from blood and bone marrow. *Scand. J. Clin. Invest.*, **21**(suppl.), 97.

CLAPHAM, P., NAGY, K., CHEINGSONG-POPOV, R., EXLEY, M. & WEISS, R.A. (1983). Productive infection and cell-free transmission of human T-cell leukemia/lymphoma virus in a non-lymphoid cell line. *Science*, **222**, 1125.

COLLINS, S.J., GALLO, R.C. & GALLAGHER, R.E. (1977). Continuous growth and differentiation of human myeloid leukaemic cells in suspension culture. *Nature*, **270**, 347.

DE VECCHIS, L., GRAZIANI, G., MACCHI, B. & 5 others (1985). Decline of natural cytotoxicity of human lymphocytes following infection with human T-cell leukaemia/lymphoma virus (HTLV). *Leuk. Res.*, **9**, 349.

GALLO, R.C. (1985). The human T-cell leukemia/lymphotropic retroviruses (HTLV) family: Past, present and future. *Cancer Res.*, **45**, 4524s.

GRAZIANI, G., PASQUALETTI, D., LOPEZ, E. & 5 others (1987). Increased susceptibility of peripheral mononuclear cells of leukemic patients to HTLV-I infection *in vitro*. *Blood*, **69**, 1175.

HASELTINE, W.A., SODROSKI, J. & ROSEN, C. (1985). The Lor gene and the pathogenesis of HTLV-I, II and III. *Cancer Res.*, **45**, 4545s.

HATTORI, T., UCHIYAMA, T., TOIBANA, T., TAKATSUKI, K. & UCHINO, H. (1981). Surface phenotypes of Japanese adult T-cell leukemia cells characterized by monoclonal antibodies. *Blood*, **58**, 645.

HERBERMANN, R.B., AOKI, T., NUNN, M. & 5 others (1974). Specificity of $^{51}$Cromium-release cytotoxicity of lymphocytes immune to murine sarcoma virus. *J. Natl Cancer Inst.*, **53**, 1103.

HO, D.D., ROTA, T.R. & HIRSCH, M.S. (1984). Infection of human endothelial cells by human T-lymphotropic virus type I. *Proc. Natl Acad. Sci. USA*, **81**, 7588.

HOLT, P.G., WARNER, L.A. & MAYRHOFER, G. (1981). Macrophages as effectors of T suppression: T-lymphocyte-dependent macrophage-mediated suppression of mitogen-induced blastogenesis in the rat. *Cell. Immunol.*, **63**, 57.

HOXIE, J.A., MATTHEWS, D.M. & CINES, D.B. (1984). Infection of human endothelial cells by human T-cell leukemia virus type I. *Proc. Natl Acad. Sci. USA*, **81**, 7591.

HULEIHEL, M. & ABOUD, M. (1983). Inhibition of retrovirus DNA supercoiling in interferon-treated cells. *J. Virol.*, **48**, 120.

KAFATOS, F.C., WELDON JONES, C. & EFSTRATIADIS, A. (1979). Determination of nucleic acid sequence homologies and relative concentrations by a dot hybridization procedure. *Nucleic Acid Res.*, **7**, 1541.

LENGYEL, P. (1982). Biochemistry of interferons and their actions. *Ann. Rev. Biochem.*, **51**, 251.

LONGO, D.L., GELMANN, E.P., COSSMANN, J. & 4 others (1984). Isolation of HTLV-I transformed B-lymphocyte clone from a patient with HTLV-associated adult T-cell leukemia. *Nature*, **310**, 505.

LOZZIO, C.B. & LOZZIO, C.B. (1975). Human chronic myelogenous leukemia cell line with positive Philadelphia chromosome. *Blood*, **45**, 321.

MACCHI, B., POPOVIC, M., ALLAVENA, P. & 4 others (1987). *In vitro* susceptibility of different human T-cell subpopulations and resistance of large granular lymphocytes to HTLV-I infection. *Int. J. Cancer*, **40**, 1987.

MARKHAM, P.P., SALAHUDDIN, S.Z., MACCHI, B., ROBERT-GUROFF, M. & GALLO, R.C. (1984). Transformation of different phenotypic types of human bone marrow T lymphocytes by HTLV-I. *Int. J. Cancer*, **33**, 13.

MERL, S., KLOSTER, B., MOORE, J. & 9 others (1984). Efficient transformation of previously activated and dividing T lymphocytes by human T-cell leukemia lymphoma virus. *Blood*, **64**, 967.

MINKS, M.A., BENVIN, S., MARONEY, P.A. & BAGLIONI, C. (1979). Synthesis of 2′5′-Oligo (A) in extracts from interferon-treated HeLa cells. *J. Biol. Chem.*, **254**, 5058.

MINOWADA, J., OHNUMA, T. & MOORE, G.E. (1982). Rosette-forming human lymphoid cell lines. I. Establishment and evidence for origin of thymus-derived lymphocytes. *J. Natl Cancer Inst.*, **49**, 891.

MIYOSHI, I., KUBONISHI, I., YOSHIMOTO, S. & 7 others (1981). Detection of type C virus particles in a cord blood T-cell line derived by cocultivation of normal human lymphocytes and human leukemic T-cells. *Nature*, **296**, 770.

MORGAN, D.A., RUSCETTI, F.W. & GALLO, R.C. (1976). Selective *in vitro* growth of T-lymphocytes from normal human bone marrow. *Science*, **193**, 1007.

NAIR, M.P.N., SCHWARTZ, S.A. & MENON, M. (1985). Association of decreased natural and antibody dependent cellular cytotoxicity and production of natural killer cytotoxic factor and interferon in neonates. *Cell. Immunol.*, **94**, 159.

PERUCHO, M. & ESTEBAN, M. (1985). Inhibitory effect of interferon on the genetic and oncogenic transformation by viral and cellular genes. *J. Virol.*, **54**, 229.

POPOVIC, M., FLOMENBERG, N., VOLKMAN, D.J. & 4 others (1984). Alteration of T-cell functions by infection with HTLV-I or HTLV-II. *Science*, **226**, 459.

POPOVIC, M., LANGE-WANTZIN, G., SARIN, P.S., MANN, D. & GALLO, R.C. (1983*a*). Transformation of human umbilical cord blood T-cells by human T-cell leukemia/lymphoma virus. *Proc. Natl Acad. Sci. USA*, **80**, 5402.

POPOVIC, M., SARIN, P.S., ROBERT-GUROFF, M. & 4 others (1983*b*). Isolation and transmission of human retrovirus (human T-cell leukemia virus). *Science*, **219**, 856.

ROBERT-GUROFF, M., RUSCETTI, F.W., POSNER, B.J. & GALLO, R.C. (1981). Detection of the human T-cell lymphoma virus p19 in cells of some patients with cutaneous T-cell lymphoma and leukemia using a monoclonal antibody. *J. Exp. Med.*, **154**, 1957.

RUSCETTI, F.W., MIKOVITS, J.A., KLAYANARAMAN, V.S. & 6 others (1986). Analysis of effector mechanisms against HTLV-I and HTLV-II/LAV infected lymphoid cells. *J. Immunol.*, **136**, 3619.

SCOTT, G.M. (1983). The antiviral effects of interferon. In *Interferons: From molecular biology to clinical application*, Burke, D.C. & Morris, A.G. (eds) p. 276. Cambridge University Press: Cambridge.

SINANGIL, F., HARADA, S., PURTILO, D.T. & VOLSKY, D.J. (1985). Host cell range of adult T-cell leukemia virus. I. Viral infectivity and binding to various cells as detected by flow cytometry. *Int. J. Cancer*, **36**, 191.

TAKATSUKI, K., YAMAGUCHI, K., KAWANO, F. & 6 others (1985). Clinical diversity in adult T-cell leukemia/lymphoma. *Cancer Res.*, **45**, 4644s.

THORN, R.M. & HENNEY, C.S. (1976). Kinetic analysis of target cell destruction by effector T-cells. I. Delineation of parameters related to the frequency and lytic efficiency of killer cells. *J. Immunol.*, **117**, 2213.

TOMITA, S., AMBRUS, J.L., VOLKMAN, D.J. & 4 others (1985). Human T-cell leukemia/lymphoma virus I infection and cloning of normal human B-cells. *J. Exp. Med.*, **162**, 393.

VAN FURTH, R. (1982). Current view on the mononuclear phagocyte system. *Immunology*, **161**, 178.

VEIT, B.C. (1982). Immunoregulatory activity of culture-induced suppressor macrophages. *Cell. Immunol.*, **72**, 14.

VITIELLO, A., MACCARIO, R., MONTAGNA, D. & 6 others (1984). Lymphocyte subpopulations in the neonate: A subset of HNK-1, OKT3, OKT8 lymphocytes displays natural killer activity. *Cell Immunol.*, **85**, 252.

VOLKMAN, D.J., POPOVIC, M., GALLO, R.C. & FAUCI, A.S. (1985). Human T-cell leukemia/lymphoma virus-infected antigen-specific T-cell clones: Indiscriminant helper function and lymphokine production. *J. Immunol.*, **134**, 4237.

WALDMANN, T.A., GREENE, W.C., SARIN, P.S. & 10 others (1984). Functional and phenotypic comparison of human T-cell leukemia/lymphoma virus negative Sezary leukemia and their distintion using anti-Tac monoclonal antibody identifying the human receptor for T-cell growth factor. *J. Clin. Invest.*, **73**, 711.

WEISS, R. (1984). Tissue specific transformation by human T-cell leukemia virus. *Nature*, **310**, 273.

YARCHOAN, R., GUO, H.G., REITZ, M.S., MALUISH, A., MITSUYA, H. & BRODER, S. (1986). Alterations in cytotoxic and helper T-cell function after infection of T-cell clones with human T-cell leukemia virus type I. *J. Clin. Invest.*, **77**, 1466.

YOSHIDA, M., MIYOSHI, I. & HINUMA, Y. (1982). Isolation and characterization of retrovirus from cell lines of human adult T-cell leukemia and its implication in the disease. *Proc. Natl Acad. Sci. USA*, **79**, 2031.